# Tritium-Labeled Compounds X. Isotope Effects in the Oxidation of Aldoses-*1-t* With Bromine

**DOI:** 10.6028/jres.068A.013

**Published:** 1964-04-01

**Authors:** Horace S. Isbell, Lorna T. Sniegoski

## Abstract

Tritium isotope effects have been used in bromine oxidation studies of aldoses to evaluate two reaction paths previously postulated by Isbell and Pigman, namely, direct oxidation and oxidation after anomerization. Because the former path involves a primary isotope effect (k*/k = 0.14, approximately) and the latter a secondary isotope effect (k*/*k* = 0.80, approximately) it was found possible to evaluate the relative importance of the two reaction paths from the overall isotope effects, which ranged from 0.20 to 0.59.

Under the conditions reported, the direct oxidation for the axial anomers ranges from 38 percent for *α*-d-galactose to 94 percent for *α*-d-lyxose. Differences in the proportion of the anomer oxidized by each of the two paths are explained by variations in the free energy required for reaching the respective transition states. Aldoses of high conformational stability having an axial C1-hydroxyl group resist the change in conformation necessary for direct oxidation and react in large measure through a change in configuration (anomerization). Aldoses of lower conformational stability having an axial C1-hydroxyl group react largely through a change in conformation, because this path does not have a high energy barrier.

The equatorial anomers of d-glucose-*1*-*t* and maltose-*1*-*t* showed isotope effects of 0.32 and 0.23, respectively in comparison with a value of 0.14 previously found for the oxidation of aldoses-*1-t* with iodine in alkaline solution. The isotope effect in the oxidation of d-glucose-*1-t* with d-glucose oxidase at 30 °C was found to be 0.15. These strong isotope effects are in accord with the rupture of the C1 to H bond in the rate-determining step.

The following reactions gave the values for k*/k cited in parentheses: d-glucose-*1-t +*NaCN (0.83); d-glucose-*1*-*C*^14^ + NaCN (1.00); d-glucose-*1-t+*NaBH_4_ (0.73); d-mannose-*1*-*t* + phenylhydrazine (0.83); and d-mannose-*1*-*C*^14^ + phenylhydrazine (0.95). The values of k*/k are typical of aldose reactions involving rate-determining isomerization of the pyranose structure.

The isotope effects were measured by a double-label method and by a newly developed method based on the radioassay of water evolved in the reaction.

## 1. Introduction and Discussion

Certain reactions take place by two or more paths having different rate-determining steps which show different isotope effects. Under suitable conditions, the size of the isotope effect can serve as a measure of the relative importance of the paths. This property has now been used in studying the mechanisms of oxidation of aldoses with bromine.

Early work in this laboratory [1, 2]^1^ showed that the ring forms of aldoses are oxidized by bromine to lactones without cleavage of the ring, and that there are wide differences in the rates of oxidation for the various aldoses and for the *α* and *β* anomers of a single aldose. It was found [3, 4] that, in the presence of a large excess of free bromine, *“β* aldoses” are oxidized rapidly, at a rate proportional to the concentration of free bromine, whereas *“α* aldoses” are oxidized slowly. Isbell and Pigman [3, p. 349] considered the oxidation of *α*-d-glucose to consist of “two reactions, namely, the oxidation of the alpha form directly, and the conversion of the alpha to the beta form with the subsequent rapid oxidation thereof.” They estimated that about 80 percent of the overall oxidation of *α*-d-glucose takes place *directly*, in solutions saturated with bromine and buffered with barium carbonate and carbon dioxide. The rate constants which they determined for the overall oxidation of eighteen *α* and *β* aldoses [4, 5, pp. 436 and 455] showed a correlation of reaction rate, structure and configuration which Isbell considered to be evidence regarding the conformation of the particular pyranose ring [6, p. 523].

Later, Barker, Overend, and Rees [7] concluded that the rate-determining step in the oxidation of the *α* aldoses studied by Isbell and Pigman consists very largely of anomerization to the corresponding *β* aldoses, and that direct oxidation of the *α* aldoses is of little importance. Thereafter, Isbell reviewed the early work and interpreted the results in the light of present concepts of reaction mechanism and conformation [8, 9].

Because mutarotation and oxidation reactions take place concurrently, quantitative evaluation of the paths by conventional methods is not satisfactory. Hence, an independent method was needed for ascertaining the relative importance of the two paths.

Isotope effects 2 provide a powerful means for distinguishing between reaction mechanisms. In general, the isotope effect is large when the isotopic bond is ruptured in the rate-determining step and small when this bond is not involved [10, p. 88]. In the reaction of an aldose with bromine, the Cl to H bond is ruptured in the oxidation step but not in the anomerization step. Hence, it seemed probable that kinetic isotope-effects of tritium could provide a means for distinguishing between direct oxidation and oxidation after anomerization.

Previously, Friedberg and Kaplan [11] had reported isotope effects^3^ of 0.2 and 0.25, respectively, for the oxidation of *α*-d-glucose-*1*-*t* and *β*-d-glucose-*1-t* with bromine in solutions buffered with barium carbonate. From the magnitude of the isotope effects, they concluded that the C1 to H bond is ruptured in the rate-determining step. They noted that the oxidation of *α*-d-glucose is complicated by the anomerization reaction, but did not pursue the subject further.

In the present investigation, isotope effects were measured for the oxidation of a group of aldoses-*1-t* with bromine in solutions buffered with sodium benzoate and benzoic acid.^4^ For each aldose, the relative importance of the two reaction paths was estimated from the observed isotope effect as compared with isotope effects characteristic of reactions in which the rate-determining step is known to be (1) cleavage of the C1 to H bond and (2) anomerization. Thus, if $k_{1}^{*}/k_{1}$ is the isotope effect for path (1) and $k_{2}^{*}/k_{2}$ for path (2), it follows that the isotope effect for the overall reaction is

$$k^{*}/k=\left(k_{1}^{*}+k_{2}^{*}\right)/\left(k_{1}+k_{2}\right)$$ (1)

and that the ratio of the rates of reaction, for the nonisotopic molecules, by the two paths is

$$k_{1}/k_{2}=\left(k_{2}^{*}/k_{2}-k*/k\right)/\left(k*/k-{k_{1}^{*}/k}_{1}\right).$$ (2)

The value used for *k*_1_^*^/k_1_ (0.14) was obtained from measurements of the oxidation of aldoses-*1*-*t* with iodine in alkaline solution [12]. This reaction is considered typical of those in which the rate-determining step involves cleavage of the C1 to H bond.

The value used for *k*_2_*/k_2_ (0.80) is the average of the results reported for aldoses-*1*-*t* in table 1 (starred values). Each of the reactions yields an open-chain product by way of a rate-determining anomerization. By assuming the above values for *k*_1_^*^/*k*_1_ and *k*_2_*/k_2_, the ratios of the rates by the two paths, *k*_1_*/k*_2_, were calculated by eq (2) from the observed, overall isotope effects.

Table 2 presents a summary of the results obtained for nine of the ten aldoses which Barker, Overend, and Rees had concluded, from the data of Isbell and Pigman, are oxidized by bromine predominantly by way of a rate-determining anomerization. Our results show that the direct oxidation ranges from 38 percent for *α*-d-galactose to 94 percent for *α*-d-lyxose.

In each instance, the extent of the reaction that takes place by way of anomerization parallels the conformational stability of the anomer. Aldoses which have an axial hydroxyl group at C1 in a highly stable conformation (e.g., *α*-d-glucopyranose-CA^5^ and *α*-d-galactopyranose-CA), resist the change in conformation necessary for direct oxidation, and react largely through anomerization (change in configuration). Aldoses of lower conformational stability (e.g., *α*-d-lyxopyranose-CA, *α*-d-talopyranose-CA, and *α*-d-mannopyranose-CA) react chiefly by a change in conformation without a change in configuration.

The rate of the direct reaction depends on the difference in free energy between the reactants in the ground state and in a transition state in which the anomeric oxygen atom lies in the same plane as C1, C2, C5, and the ring oxygen atom [9]. The free energy required in order to reach this transition state depends on the structure and the configuration. With molecules having an axial hydroxyl group at C1 in a highly stable conformation, much energy is required for so changing the conformation as to bring the hydroxyl group into an equatorial position. *α*-d-Glucopyranose and *α*-d-galactopyranose have high stability in that chair conformation having an axial hydroxyl group at C1; this fact accounts for the low rates of oxidation and the tendency of these sugars to react through a change in configuration rather than conformation.

The higher reactivity of sugars having the *α*-d-*manno* structure is in accord with the fact that, in the transition state of the *manno* group, the C1-hydroxyl group is eclipsed by the C2-hydrogen atom (instead of by the larger C2-hydroxyl group, as with *α*-d-glucopyranose). The high reactivity of the axial anomers of pentopyranoses, in comparison with the axial anomers of hexopyranoses can be explained by a lower energy-requirement for a change in conformation in the pentopyranose series; this arises from the lack of a large substituent group at C5 which, in the transition state of a hexopyranose, must assume an axial position. The high isotope-effect for *α*-d-lyxopyranose-*1-t*, and the small difference in the rates of reaction for the *a* and *β* anomers of this aldose, are in accord with only a small difference in the relative stabilities of the CA and CE conformations of this aldose [14].

Table 3 presents results obtained in the individual measurements summarized in table 2. It includes, in addition, measurements of k*/k for the bromine oxidation of *β*-maltose-*1-t.* This sugar was selected for ascertaining the isotope effect in the bromine oxidation of an aldose having an equatorial hydroxyl group, because it is one of the few sugars that crystallize only in this form. The value of k*/k obtained (0.23) is somewhat high, but is sufficiently close to the expected value for the primary isotope effect (0.14) to indicate cleavage of the C1 to H bond in the rate-determining step.

Interpretation of the reactions of the *α* and *β* aldopyranoses depends on the correct assignment of configuration to the anomers. Recent papers by Blom [15] and Christiansen [16] challenged the generally accepted assignments of configuration for the *α* and *β* aldoses. This concept has now been shown to be erroneous [17]. One of Blom’s reasons for questioning the validity of the assignments is that the aldoses ordinarily considered to have equatorial C1-hydroxyl groups are oxidized more *rapidly* than those having axial C1-hydroxyl groups, whereas cyclohexane derivatives having equatorial hydroxyl groups are oxidized more *slowly* than those having axial hydroxyl groups. As pointed out earlier [9], differences in the relative rates of oxidation of the axial and equatorial derivatives of cyclohexane and of pyranoses can be ascribed to the presence or absence of the ring oxygen atom. With aldopyranoses, the ring oxygen atom provides a low-energy path for the reaction by furnishing electrons in a transition state favored by resonance. This path requires a transition state in which the ring oxygen atom, the hydroxyl oxygen atom, and C1, C2, and C5 lie in one plane. The large differences in the rates of reaction for the *α* and *β* aldopyranoses arise from variation in the free energy required in order that the compounds may attain this planar transition state. With a derivative of cyclohexane, there is no resonance stabilization of a planar transition state and, consequently, this path is not favored. The absence of this low-energy path accounts for the lower rates of oxidation of cyclohexane derivatives and the lack of appreciable difference between the rates of reaction of the axial and the equatorial derivatives of 4-*tert*-butylcyclohexanol [18].

## 2. Experimental Details

### 2.1. Materials

The labeled aldoses listed below were prepared by the methods given in the references cited: Aldoses-*1-t* [19], d-glucose-*1-C*^14^ [20], d-glucose-*6-C*^14^ [21], d-glucose-*6-t* [22], d-mannose-*1-C*^14^ [20], d-mannose-*2-C*^14^ [23], and d-mannose-*6-t* [24]. The radioactivity of each tritium-labeled aldose was adjusted, by addition of a carrier, to about 1 *μ*c/mg. The doubly labeled aldoses, d-glucose-*1-t*,*6-C*^14^, d-glucose-*1-C*^14^,*6-t*, d-mannose-*1-t*, *2-C*^14^, and d-mannose-*1-C*^14^, *6-t* were prepared by crystallizing mixtures of the singly labeled aldoses containing, respectively, from 1 to 2 *μ*c/mg of tritium and 0.1 to 0.2 *μ*c/mg of carbon 14. The purity of each labeled aldose was checked by paper chromatography.

The buffered bromine solution used for the oxidation measurements contained 0.90 g of benzoic acid, 2.16 g of sodium benzoate, and 3 ml of bromine (an excess) in 100 ml of aqueous solution.

### 2.2. General Procedures

#### a. Determination of Aldoses in Reaction Mixtures

The Somogyi method [5, p. 196] was used for determination of the residual aldoses in the reaction mixtures, by selecting suitable heating times and by standardizing with known quantities of the various aldoses. The heating times and factors for converting the titrations to a weight basis are given in table 4. The factors were derived from control measurements run in duplicate with known weights of each aldose.

#### b. Determination of Carbon 14 and Tritium

Carbon-14 and tritium were determined either by counting in solution [25] with a Packard Tri-Carb Liquid Scintillation Spectrometer or by counting in films [26, 27] with a windowless, gas-flow, proportional counter. The solution used for scintillation counting contained, per liter, 7 g of 2,5-diphenyloxazole (PPO, scintillation grade), 0.3 g of 2,2′- *p*- phenylenebis(4 - methyl- 5 - phenyloxazole) (dimethyl-POPOP, scintillation grade), and 100 g of naphthalene, in dioxane (Eastman). The procedure for counting doubly labeled materials in the scintillation counter is described in [12].

The *O*-(carboxymethyl) cellulose (CMC) solution (used for preparing films for the assay of radioactivity with the proportional counter) contained 1.5 g of CMC (medium viscosity CMC, product of Hercules Powder Co.), 0.2 g of phenol, and 1 ml of a red ink (eosin type), dissolved in sufficient water to yield 100 ml of solution. The films were prepared and counted as described in [26, 27].

### 2.3. Measurements of k*/k for the Oxidation of Aldoses-*1*-*t* With Bromine

#### a. Water-*t* Method [12]

Samples of aldoses-*1*-*t* of known weight (about 0.05 mmole) were placed in test tubes (20 × 150 mm). Each tube was suspended in an ice-water bath and two ml of ice-cold, buffered bromine solution (sec. 2.1) was added. The tube was stoppered, and mechanically shaken in the bath for the time given in table 3. Then, about 10 ml of ice-cold, distilled water was added, and the excess bromine was removed by bubbling ethylene through the solution for about 1 min. The bromine-free solution was quantitatively transferred to a volumetric flask, the volume was made to 25 ml, and aliquots were taken for determining the reducing sugar (residual reactant) and the water-*t*.

Measurements of reducing sugar were made with two 2-ml and two 4-ml aliquots of the bromine-free solution. Each aliquot was diluted to 5 ml and analyzed by the procedure described in [5, p. 196], using the heating times and factors given in table 5. For each measurement, the results from the 4 aliquots were averaged, and the extent of the oxidation (*f* of eq (3)) was calculated.

For determination of water-*t*, a 2-ml aliquot of the bromine-free solution was transferred to a flask (round-bottomed, standard-tapered, inner-joint) containing about 50 mg of sodium carbonate, and the water-*t* was sublimed by the procedure described in [12]. The water-*t* was analyzed in duplicate with the liquid scintillation counter by use of 100-*μ*l samples and 10-ml aliquots of scintillator solution. Each sample was counted to a total of 10,000 counts, to give a statistical accuracy of ±1 percent.

The isotope effect, k*/k, was calculated by the following equation [28, 29]:

k*/k=log(1−rf)/log(1−f) (3)

where *f* is the fraction of initial reactant oxidized, (1—*f*) is the fraction of initial reactant remaining, and *rf* is the ratio of the total activity (*μ*c) in the tritiated water formed to the activity (*μc*) of tritium in the initial reactant (calculated from the weight and specific activity of the aldose-*1*-*t* used).

#### b. Double-Label Method [26]

Measurements of k*/k were made for the oxidation of *α*-d-glucose-*1*-*t* and *α*-d-mannose-*1*-*t* by the double-label method, with carbon 14 as the reference isotope (table 5). In each instance, the isotope effect was calculated from the equation:

k*/k=1+[log(p/p°)/log(1−f)] (4)

where *p°* is the ratio of the functional to the reference isotope in the initial reactant, and *p* is the ratio of the same isotopes in the residual reactant.

Samples (about 9 mg) of the doubly labeled aldoses were weighed, by means of a semimicro balance, into separate test tubes. Each tube was placed in an ice-water bath, and 2 ml of ice-cold, buffered bromine solution was added. The tube was stoppered, and was mechanically shaken in the bath for a time sufficient for 50 to 90 percent of the aldose to become oxidized. Then, the reaction was stopped by dilution with water containing 100 mg of the nonradioactive aldose (as carrier) and 200 mg of decolorizing carbon. The mixture was filtered, t he bromine-free filtrate was passed through 15 ml of mixed anion- and cation-exchange resins, and the salt-free effluent was concentrated. The resulting sirup was diluted with a few drops of methanol, and 2-propanol was added to incipient turbidity. The crystals that formed were separated, and recrystallized twice from water, with addition of methanol and 2-propanol. The purified mixture of the residual reactant and carrier was analyzed for carbon 14 and tritium, and the ratio of tritium to carbon 14 in the residual reactant (*p* of eq (4)) was calculated.

The ratio of tritium to carbon 14 in the initial reactant (*p°* of eq (4)) was determined from a sample of the original reactant after dilution with carrier to about the same level of activity as the samples derived from the oxidation mixture.

If *w*_0_ is the weight (in mg) of initial reactant (having *s* activity of the reference isotope per mg), *w_r_* the weight of residual reactant, $w_{r}^{\prime }$ the weight of nonradioactive (reactant) carrier added, and *s*′ the activity of the reference isotope per mg of the residual reactant-carrier mixture, then

$$w_{r}=s\prime w_{r}^{\prime }/\left(s-s\prime \right),$$ (5)

and the fraction of reactant oxidized is

$$f=\left(w_{0}+w_{r}\right)/w_{0}=\left[w_{0}\left(s-s\prime \right)-w\prime_{r}s\prime \right]/w_{0}(s-s\prime ).$$ (6)

Table 5 is a résumé of measurements of k*/k for the oxidation of aldoses-*1-t* (with carbon-14 as the reference isotope) and of aldoses-*1*-*C*^14^ (with tritium as the reference isotope). The values of k*/k obtained for the oxidation of *α*-d-glucose-*1*-*t*,*6*-*C*^14^ and *α*-d-mannose-*1*-*t*,*2*-*C*^14^ by the double-label method (0.59 and 0.41, respectively) are somewhat higher than the values (0.53 and 0.30) obtained by the water-*t* method. Determinations are based on the first part of the reaction in the water-*t* method and on the last part in the double-label method.

### 2.4. Measurements of k*/k for Reactions Involving Rate-Determining Isomerization

#### a. Reaction of d-Glucose-*1*-*t*,*6*-*C*^14^ With Sodium Cyanide

Samples of about 0.05 mmole of d-glucose-*1*-*t*,*6*-*C*^14^ were weighed into small test tubes. Each sample was dissolved in 100 *μ*l of water, and sufficient 0.47 *M* sodium cyanide in 0.50 *M* sodium hydroxide was added to combine with 50 to 70 percent of the d-glucose-*1*-*t*,*6*-*C*^14^. The tubes were sealed, and allowed to stand at 25 °C for 24 hr and in the freezer for several days. The tubes were opened, and 100 mg of d-glucose was added to each. After the addition of a few drops of 10-percent, aqueous formic acid, each solution was evaporated to a sirup (to remove excess hydrogen cyanide). The sirup was dissolved in water, and the solution was passed through a column of mixed anion- and cation-exchange resins to remove all ionic substances. The effluent was concentrated, and the d-glucose-*1*-*t*,*6*-*C*^14^ was separated by crystallization. The material was recrystallized twice from water, with addition of methanol and 2-propanol, dried, and radioassayed on planchets for carbon 14 and tritium. The ratio of tritium to carbon 14 (*p* of eq (4)) was calculated, as well as the extent of reaction (*f* of eq (6)). The ratio of tritium to carbon 14 in the initial reactant (*p°* of eq (4)) was determined from a sample of the original reactant after dilution with carrier to about the same level of activity as that of the samples derived from the oxidation mixture. k*/k was then calculated from eq (4). The results are reported in table 1.

The method was also applied to a study of the reaction of d-glucose-*1-C^14^,6-t* with sodium cyanide. In this instance, tritium was used as the reference isotope for the study of the effect of carbon 14 at C1.

#### b. Reduction of d-Glucose-*1*-*t*,*6*-*C*^14^ With Sodium Borohydride

A 9-mg sample of d-glucose-*1*-*t*,*6*-*C*^14^ was weighed into a small test tube, and a solution containing 0.5 mg of sodium borohydride in 50 *μ*l of water was added. The reaction mixture was kept in an ice-water bath for 30 to 40 min and at room temperature for 30 min. Then, a drop of glacial acetic acid, 98.2 mg of d-glucitol carrier, and 5 ml of water were added. The solution was passed through several ml of a cation-exchange resin, and the effluent was concentrated under diminished pressure to a sirup. Methanol was added, and the solution was concentrated; this process was repeated several times. The d-glucitol-*1*-*t*,*6*-*C*^14^ in the residue was recrystallized three times from pyridine, with addition of acetone. The pyridine complex was decomposed by heating to constant weight at 85 °C in a vacuum oven. The pure product-carrier mixture and the initial reactant were assayed in films, on planchets, for carbon 14 and tritium.

The weight of product was calculated from the activity of the product-carrier mixture and that of the initial reactant by the equation:

$$w_{p}=s\prime w_{p}^{\prime }/\left(s-s\prime \right)$$ (7)

where *w_p_* is the weight (in mg) of product (having *s* activity of the reference isotope per mg), $w_{p}^{\prime }$ the weight of nonradioactive carrier added, and *s*′ the activity of the reference isotope per mg of product-carrier mixture. Then

$$f=w_{p}m_{r}/w_{0}m_{p}=w_{p}^{\prime }s\prime m_{r}/w_{0}\left(s-s\prime \right)m_{p}$$ (8)

where *m_r_* and *m_p_* are the equivalent weights of the reactant and product, respectively.

The value of k*/k was calculated from the eq [26]:

k*/k=log(1−fp′/p°)/log(1−f) (9)

where *p°* is the ratio of tritium to carbon 14 in the initial reactant, and *p*′ is the ratio in the product.

#### c. Formation of d-Mannose-*1*-*t*,*2*-*C*^14^ Phenylhydrazone

A 54-mg sample (0.3 mmole) of d-mannose-*1*-*t*,*2-C^14^* was weighed into a small test tube. Then, 0.9 ml of 0.6-percent acetic acid was added, and the solution was allowed to stand for 18 to 20 hr. Sufficient 0.251-*M* phenylhydrazine in 0.6-percent aqueous acetic acid to give a 20- to 30-percent reaction (0.24 to 0.36 ml) was added, and the mixture was allowed to stand at 25 °C for 1 hr with occasional shaking. The resulting labeled d-mannose phenylhydrazone was removed by filtration, successively washed with about 2 ml each of water, ethanol, and ether, and dried overnight in a vacuum desiccator.

d-Mannose-*1*-*t* phenylhydrazone shows an isotope effect on recrystallization [30]; hence, for analysis, the d-mannose-*1*-*t*,*2*-*C*^14^ phenylhydrazone was reconverted to d-mannose-*1*-*t*,*2*-*C*^14^. There is no isotope effect in this conversion because the reaction goes to completion. The labeled d-mannose phenylhydrazone was transferred to a 100-ml, standard-tapered flask, and 300 mg of nonradioactive d-mannose phenylhydrazone carrier, a stirring bar, 8 ml of distilled water, and 155.9 *μ*l (1.5 mmole) of benzaldehyde were added. The mixture was heated, with stirring, in a boiling-water bath for 30 min; the appearance of the crystals changed rapidly. Additional water was added to rinse down the sides of the flask. The mixture was cooled, and the benzaldehyde phenylhydrazone was removed by filtration. The filtrate was extracted five times with ether to remove benzaldehyde. The solution was then passed through 10 ml of mixed anion- and cation-exchange resins and concentrated under reduced pressure on a rotary evaporator. Water was added, and the concentration was repeated. The sirup was dissolved in methanol, and the solution was filtered into a test tube and concentrated to a sirup from which d-mannose-*1-t,2-C*^14^ crystallized after addition of methanol and 2-propanol. The material was crystallized three times, dried, and assayed (in films on planchets) for carbon 14 and tritium. *w_p_* was calculated from eq (7) with *w_p_* and $w_{p}^{\prime }$ the weights of d-*mannose* in the product and nonradioactive carrier, respectively. Then,

$$f=w_{p}/w_{0}.$$ (10)

The isotope effect, k*/k, was then calculated from eq (9).

The results are reported in table 1, and, in addition, those for the isotope effect of carbon 14 in the formation of d-mannose-*1*-*C*^14^,*6*-*t* phenylhydrazone from d-mannose-*1*-*C*^14^,*6*-*t*, in which reaction tritium was the reference isotope.

### 2.5. Measurements of k*/k for the Oxidation of d-Glucose-*1*-*t* and d-Glucose-*1*-*C*^14^ With d-Glucose Oxidase by the Double-Label Method

Samples of either d-glucose-*1*-*t*,*6*-*C*^14^ or d-glucose-*1-C*^14^,*6-t* of known weight (about 10 mg) and specific activity were placed in test tubes (20×150 mm). After addition of 2 ml of an aqueous solution containing 430 mg of disodium hydrogen phosphate dodecahydrate, 84 mg of citric acid, and 2 mg of d-glucose oxidase,^6^ each tube was stoppered and mechanically shaken in a water bath at 30 °C for the time given in table 6. Then the reaction mixture was cooled in an ice bath, diluted with a solution of 100 mg of d-glucose carrier in 5 ml of water and passed through a column containing 15 ml of mixed anion- and cation-exchange resins. The combined effluent and wash liquors were concentrated in a rotary vacuum still to about 2 ml and filtered after addition of decolorizing carbon. The solution was collected in a test tube and concentrated to a sirup in a stream of dry air. The sirup was diluted with 2 ml of methanol and again concentrated; addition and evaporation of methanol were repeated twice more. The residual sirup was dissolved in 5 drops of methanol, and 2-propanol was added to the point of incipient turbidity; *α*-d-glucose crystallized from the solution. After one day the mother liquor was removed with a capillary pipet, and the crystals were washed twice with a 1:1 mixture of methanol and 2-propanol. The crystals were recrystallized three times from aqueous methanol as just described. The resulting product was dried to constant weight and analyzed for carbon 14 and tritium by the method previously described [26].

For calculation of k*/k the isotope at C1 is the functional isotope. The ratio of the functional to the reference isotope in the residual reactant (*p* of eq (4)) and the extent of reaction (*f* of eq (6)), were calculated from the analytical data. The ratio of the functional to the reference isotope in the original reactant was determined on a separate sample. The measurements of k*/k are given in table 6.

## Figures and Tables

**Table 1:** Determinations of k*/k for reactions involving rate-determining isomerizations^a^

Reactant	*f*	*p°*	*p*	*p*′	k^*^/k	k^*^/k (average)

Cyanohydrin reaction						

d-Glucose-*1-t*, *6-C^14^*	0.5011	9.12	10.56		0.73	
	.5211	9.12	11.09		.79	0.76^*^
d-Glucose-*1*-*C*^14^, *6-t*	.652	0.1144	0.1144		1.00	
	.611	.1144	.1160		0.99	1.00

Sodium borohydride reduction						

d-Glucose-*1-t, 6-C^14^*	0.0874	9.12		6.74	0.73	0.73^*^

d-Mannose phenylhydrazone formation						

d-Mannose-*1*-*t*, *2-C*^14^	0.1655	8.65		7.33	0.83	
	.1672	8.65		7.27	.83	
	.2605	8.65		7.44	.84	0.83^*^
d-Mannose-*1*-*C*^14^, *6-t*	.1550	0.0668		0.0637	.95	
	.1572	.0668		.0639	.95	
	.2486	.0668		.0638	.95	.95

a Experimental details are given in section 2.4.

**Table 2:** Oxidation of aldoses with bromine (summary)

Reactant	Relative rates for overall oxidation^a^		Oxidation of axial anomer of aldose-*1*-*t*^b^		
	Axial anomer	Equatorial anomer	Isotope effect k^*^/k	Reaction paths (ratio) *k*_1_*/k*_2_^c^	Direct oxidation
					
					%
d-Galactose	1.3	50	0.55	0.6	37.5
d-Glucose	1.0	39	.53	.7	41.2
d-Mannose	1.6	24	.30	3.1	75.6
d-Talose	2.4	26	.20	10.0	90.9
l-Rhamnose	2.8	24	.25	5.0	83.3
l-Arabinose	3.0	52	.32	2.7	73.0
d-Lyxose	4.9	14	.18	15.5	93.9
d-Ribose	6.1	45	.26	4.5	81.8
d-Xylose	2.8	52	.26	4.5	81.8
Lactose	0.9	30	.46	1.0	50.0

a Measurements made at 0 °C with a barium carbonate-carbon dioxide buffer and reported by Isbell [5, pp. 436 and 455]. Based on rate of *α*-d-glucose as unity.

b Measurements made at 0 °C with a sodium benzoate-benzoic acid buffer.

c Calculated from eq (2).

**Table 3:** Experimental data for oxidation of aldoses-*1-t* with bromine

Reactant	Reaction time	*f*	*rf*	k^*^/k	k^*^/k (average)
					
	*Min*				
*α*-d-Galactose	40	0.113	0.061	0.53	
	50	.217	.130	.57	0.55
*α*-d-Glucose	45	.455	.277	.53	
	55	.402	.238	.53	.53
*α*-d-Mannose	35	.329	.108	.28	
	45	.343	.121	.31	.30
*α*-d-Talose	25	.210	.046	.20	
	25	.222	.054	.22	
	35	.289	.063	.19	
	35	.309	.073	.20	.20
*α*-l-Rhamnose H_2_O	30	.165	.046	.26	
	40	.235	.061	.24	.25
*β*-l-Arabinose	20	.151	.049	.31	
	30	.256	.094	.33	.32
*α*-d-Lyxose	10	.201	.043	.20	
	20	.326	.066	.17	.18
*β*-d-Ribose	6	.131	.037	.27	
	10	.218	.060	.25	.26
*α*-d-Xylose	20	.174	.043	.23	
	30	.258	.082	.29	.26
*α*-Lactose H_2_O	40	.454	.241	.46	
	50	.490	.268	.46	.46
*β*-Maltose H_2_O	1	.131	.033	.24	
	2	.201	.052	.24	
	3	.221	.054	.22	
	4	.326	.079	.21	.23

**Table 4:** Determination of reducing sugars by the Somogyi method

Sugar	Heating time	Aldose in sample a
		
	*min*	*mg*
l-Arabinose	30	0.133*x*+0.120
d-Galactose	30	.140*x*+.118
d-Glucose	15	.119*x*+.130
Lactose monohydrate	35	.207*x*+.250
d-Lyxose	20	.114*x*+.160
Maltose monohydrate	30	.238*x*+.265
d-Mannose	35	.118*x*+.120
l-Rhamnose monohydrate	35	.152*x*+.182
d-Ribose	25	.147*x+*.156
d-Talose	15	.143*x*+.116
d-Xylose	30	.125*x*+.080

a *x*=titer in ml of 0.005 *N* sodium thiosulfate.

**Table 5:** Determination of k*/k for oxidation of aldoses with bromine (double-label method)

Reactant	Time	*f*	*p°*	*p*	k*/k	k*/k (average)
						
	*min*					
*α*-d-Glucose-*1-t,6*-*C*^14^	180	0.495	9.12	11.92	0.61	
	300	.755	9.12	16.79	.57	0.59
*α*-d-Mannose-*1-t,2*-*C*^14^	180	.643	7.72	14.53	.39	
	240	.749	7.72	16.68	.44	
	300	.749	7.72	17.78	.40	.41
*β*-d-Glucose-*1-t,6*-*C*^14^	5	.735	9.12	22.57	.33	
	7	.710	9.12	21.01	.32	.32
*α*-d-Glucose-*1-C*^14^,*6*-*t*	180	.478	0.1144	0.1153	.99	
	300	.647	.1144	.1238	.92	.96
*β*-d-Glucose-*1-C*^14^,*6*-*t*	5	.626	.1144	.1184	.97	
	7	.700	.1144	.1227	.94	.96

**Table 6:** Determination of k*/k for oxidation of *d*-glucose with *d*-glucose oxidase (double-label method)

Reactant	Time	*f*	*p°*	*p*	k*/k	k*/k (average)
						
	*Min*					
d-Glucose-*1*-*t*, *6-C*^14^	55	0.5902	9.12	18. 64	0.20	
	65	.7384	9.12	30. 37	.10	0.15
d-Glucose-*1*-*C*^14^,*6-t*	55	.6311	0.1144	0.1211	.94	
	65	.7151	.1144	.1198	.96	

## Footnotes

1 Figures in brackets indicate the literature references at the end of this paper.

2 An isotope effect can be broadly defined as any difference between the behavior of an isotopically labeled substance and that of the nonlabeled substance. For quantitative evaluation, the effect is defined as *k***/k*, where *k** and *k* are the rates of reaction for the labeled and the nonlabeled molecules, respectively.

3 The authors actually reported values of *k/k** instead of the values of *k***/k* cited here.

4 In the early work on the oxidation of the anomeric aldoses with bromine, a buffer consisting of solid barium carbonate and carbon dioxide gas was used, because it permits study of the lactones formed. A change was made because this buffer is not suitable for tracer studies that employ small quantities of material. Both buffers maintain a slightly acid solution in which the anomerization reaction is relatively slow.

5 The system for indicating conformation is described in [13].

6 “Dee-O,” Takamine Laboratories, Clifton, N.J.
